# The Mid-Term Results of Patients who Underwent Radiofrequency Atrial Fibrillation Ablation Together with Mitral Valve Surgery

**DOI:** 10.5935/1678-9741.20160058

**Published:** 2016

**Authors:** Abdurrahim Çolak, Ugur Kaya, Munacettin Ceviz, Necip Becit, Hikmet Kocak

**Affiliations:** 1 Atatürk University, Erzurum, Turkey.

**Keywords:** Ablation Techniques, Arrhythmia, Sinus, Atrial Fibrillation

## Abstract

**Objetive:**

Saline-irrigated radiofrequency ablation, which has been widely used for surgical treatment of atrial fibrillation in recent years, is 80-90% successful in achieving sinus rhythm. In our study, our surgical experience and mid-term results in patients who underwent mitral valve surgery and left atrial radiofrequency ablation were analyzed.

**Methods:**

Forty patients (15 males, 25 females; mean age 52.05±9.9 years; range 32-74) underwent surgery for atrial fibrillation associated with mitral valvular disease. All patients manifested atrial fibrillation, which started at least six months before the surgical intervention. The majority of patients (36 patients, 90%) were in NYHA class III; 34 (85%) patients had rheumatic heart disease. In addition to mitral valve surgery and radiofrequency ablation, coronary artery bypass, DeVega tricuspid annuloplasty, left ventricular aneurysm repair, and left atrial thrombus excision were performed. Following discharge from the hospital, patients' follow-up was performed as outpatient clinic examinations and the average follow-up period of patients was 18±3 months.

**Results:**

While the incidence of sinus rhythm was 85.3% on the first postoperative day, it was 80% during discharge and 71% in the 1^st^ year follow-up examination.

**Conclusion:**

Radiofrequency ablation is an effective method when it is performed by appropriate surgical technique. Its rate for returning to sinus rhythm is as high as the rate of conventional surgical procedure.

**Table:** 

Abbreviations, acronyms & symbols	
AF	= Atrial fibrillation
ECG	= Electrocardiography
ICU	= Intensive care unit
LA	= Left atrium
NYHA	= New York Heart Association
PAP	= Pulmonary artery pressure
RF	= Radiofrequency
SR	= Sinus rhythm

## INTRODUCTION

Atrial fibrillation (AF) is an arrhythmia observed in 0.4-1% of the population, with this rate rising up to 10% in advanced ages (over 65 years of age). It has an incidence of 40-60% in patients with mitral valvular disorders, and 5-10% in patients with aortic valvular disorders^[1]^. The prevalence of AF was reported as 0.16% in the 40-59 age group, and 2.16% in patients over 60 years of age, in Turkey^[2]^.

Medical treatment may be insufficient in controlling the rate of AF, or intolerance may develop against antiarrhythmic agents, due to their side effects. Additionally, AF is known to reduce the life quality of patients due to causes such as heart failure, hemodynamic instability, palpitations, and thromboembolic events. For this reason, in patients with AF undergoing openheart surgery, surgical ablation is performed in order to provide sinus rhythm (SR) and avoid AF-related complications that may develop in the postoperative period. Surgical ablation techniques intend to achieve SR by terminating AF, together with regaining atrioventricular synchronization and atrial contractility function. The method, described by Dr. Cox, in 1980, and developed as 'Maze I' thereafter, is the gold standard in surgical treatment of AF, with a success rate of 99% today^[3,4]^. However, since the procedure is technically difficult, the cardiopulmonary bypass and operation times are long, and the risks for complications such as postoperative bleeding are high, thus, simpler and easier techniques, which are developed by using different energy sources, have gained widespread applicability. Irrigated radiofrequency ablation is one of the frequently implemented ablation methods, and it is used as monopolar and bipolar radiofrequency (RF) ablation in our clinic. In this study, our aim was to analyze our surgical experience and results in patients who had undergone mitral valve surgery and left atrial RF ablation.

## METHODS

Forty patients (15 males, 25 females; mean age 52.05±9.9; range 32-74) underwent surgery for AF associated with mitral valvular disease. All patients manifested AF, which started at least six months before the surgical intervention. In addition to mitral valve surgery and RF ablation, coronary artery bypass, DeVega tricuspid annuloplasty, left ventricular aneurysm repair, and left atrial thrombus excision were performed. Without considering performed surgical procedures, irrigated-monopolar RF ablation was used in 35 patients and irrigated-bipolar was used in five patients. The performed surgical procedures were as follows: mechanical valve replacement in 10 patients, bioprosthetic mitral valve replacement in 5 patients, mechanical mitral valve replacement and tricuspid commissurotomy in 2 patients, mechanical mitral valve replacement and left atrial thrombectomy in 3 patients, bioprosthetic mitral valve replacement and DeVega tricuspid annuloplasty and left atrial thrombectomy in 1 patient, bioprosthetic mitral valve replacement and splenectomy in 1 patient, bioprosthetic mitral valve replacement and tricuspid annuloplasty in 7 patients, mechanical mitral valve replacement and tricuspid annuloplasty in 3 patients, single-vessel coronary artery bypass and mechanical mitral valve replacement in 2 patients, triple-vessel coronary artery bypass and bioprosthetic mitral valve replacement and tricuspid annuloplasty in 1 patient, mechanical mitral valve replacement and aortic wrapping in 1 patient, mechanical mitral valve replacement and Bentall procedure in 1 patient, bioprosthetic mitral and aortic valve replacement in 1 patient, mechanical aortic and mitral valve replacement in 1 patient.

In the preoperative period, the left atrium (LA) size was measured as 53.5±6.2 mm, in average (range 33-63 mm); in seven patients, LA was 60 mm or over. The preoperative pulmonary artery pressure (PAP) was 49.8±16.4 mmHg, in average (range 30-100 mmHg); in 57 (72.2%) patients, PAP was 40 mmHg or over. In the preoperative period, EuroSCORE revealed that the standard EuroSCORE 2 value was 2.9±2.4, in average (range 0-9), and the logistic EuroSCORE 2 value was 3.1±2.1%, in average (range 0.8%-16.8%).

In one patient, mitral valve replacement and splenectomy were performed due to infective endocarditis.

**Fig 1:**
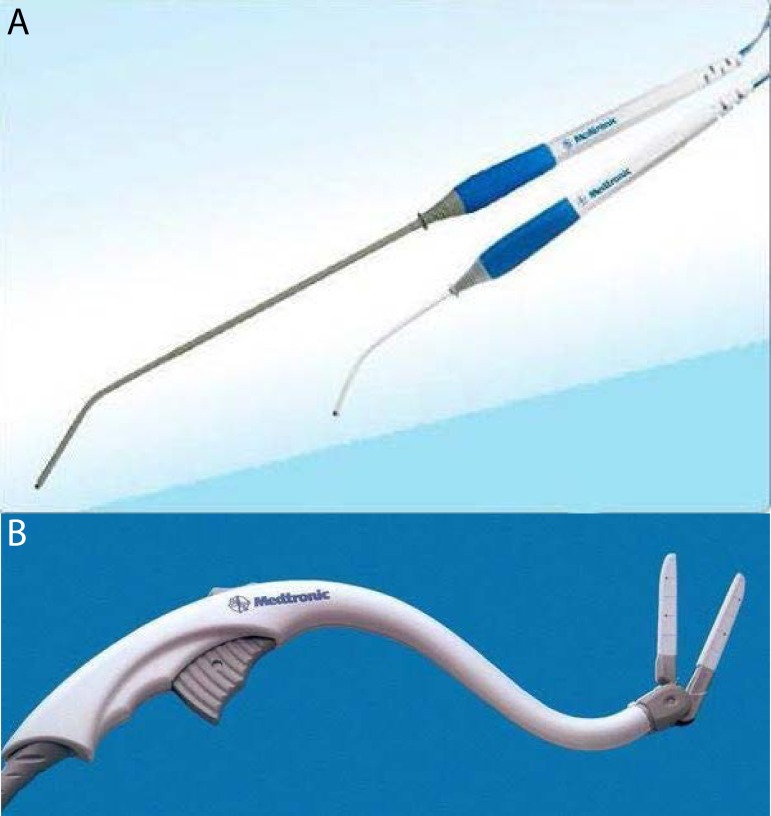
Medtronic Cardioblate™ ablation system. A: Cardioblate Surgical ablation pens. B: Cardioblate Clamp and Surgical Ablation Probe.

The unipolar ablation technique was used in the AF treatment for the patients. Following the median sternotomy, bicaval cannulation and cardiopulmonary bypass were performed. A left atriotomy was then carried out after inserting a cross-clamp. Afterwards, the process of RF was performed using the Medtronic Cardioblate^TM^ ablation system (Medtronic, Inc., Minneapolis, Minnesota, USA) composed of a power generator and an ablation clamp (Figure 1). In this study, 25 W power and 5 ml/min irrigation speed were used in the applications of unipolar RF with irrigation. The contact surface was cooled by providing irrigation through the holes on the tip of the catheter, and lesions were formed by reaching the effective ablation power in the deep tissues.

**Fig. 2:**
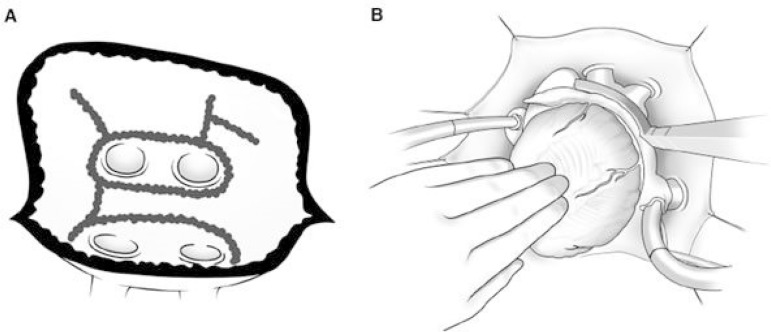
Ablation line: (A) unipolar, (B) bipolar.

In patients in whom bipolar ablation was performed simultaneously with mitral valve surgery, the system consisted of a power generator and ablation probe. The bipolar ablation technique utilizing the same ablation system was used for the patients with bipolar RF. With this system, the tip of the ablation clamp is atraumatic, and the target tissue is stabilized between the two ends. The RF energy passes through this tissue. By providing irrigation between the clamp and tissue surface simultaneously, the tissue is cooled which maintains the tissue temperature between 45 and 55°C. Furthermore, the clamp emits a signal indicating the formation of a transmural lesion by measuring the impedance between the two electrodes. This process of generator ablation ends spontaneously through this mechanism. Thus, a safe and controlled transmural ablation line is formed. This type of system can also be used with offpump and minimally invasive techniques. By fluid irrigation through the holes in the probe tip, the tissue was cooled and deeper lesions could be created. The procedure was performed through left atrial incision, anterior to the right pulmonary veins, following administration of cardioplegic arrest. After ablation was performed circumferentially from within the left atrial appendage, it was closed with sutures from inside. Then, an ablation line extending from left atrial appendage to left superior pulmonary vein was created (Figure 2). Starting from the incision made at the interatrial groove, the vicinity of right pulmonary veins was isolated semi-circumferentially with the ablation pen. Left pulmonary veins were turned around in a single circle. Then, another line, connecting two islands involving right and left pulmonary veins was created. To avoid possible esophageal injury, this line was positioned towards the left atrial roof as much as possible. Then, another ablation line, connecting left pulmonary veins and posterior annulus of mitral valve was created. A line, starting just from the midpoint of this line, was extended to the base of the atrium; with this, it was aimed to avoid reentry waves, formed by coronary sinus between atria. While ablation was done by encircling ostia of the right and left pulmonary veins, in order to prevent cascade currents, the circular area was connected from inside. The auricle of LA was sutured from inside.

In all patients, following cross-clamping, amiodarone infusion was started with a loading dose of 600-800 mg/day. In every patient, temporary epicardial pacemaker wire was placed. In patients entering AF following decannulation, internal electromechanical cardioversion was performed. In every patient, amiodarone administration, which started during the operation, continued postoperatively in the Intensive Care Unit (ICU). In two patients in whom the heart rate slowed down, amiodarone administration was terminated and temporary pacemaker support was provided. In patients in whom PR interval was prolonged in electrocardiography (ECG), the infusion dose of amiodarone was reduced. Patients who received inotropic agents for any reason (even with low-dose or short-term) were considered as supported by inotropes.

In all patients, amiodarone treatment was used for three months postoperatively. Following surgery, with the aim of rhythm control, beta-adrenergic blockers (metoprolol) or digoxin was added to the treatment of patients. The first evaluations of patients' rhythm status were made by ECG during their ICU follow-up. Following discharge from the hospital, they were followed-up by control examinations in the outpatient clinic. Patients were followed-up by ECG in the first week and in the first month. In the 3^rd^ month of the postoperative period, besides echocardiography and ECG controls, 24-hour Holter ECG was also performed. All antiarrhythmic agents were discontinued in patients who had sinus rhythm in the control examination performed in the 3^rd^ month.

### Statistical Analysis

Kaplan-Meier analysis was performed to determine the probability of survival, and survival curves were compared using the log-rank test. All values were considered to be statistically significant with a *P* value of less than 0.01.

## RESULTS

Forty patients (15 males, 25 females; mean age 52.05±9.9 years; range 32-74) underwent surgery for AF associated with mitral valvular disease. None of the patients required permanent pacemaker. The average duration of perfusion and cross-clamping time were 111±10.2 min (range 54-159 min) and 81.3±19.6 min (range 33-139 min), respectively. The average durations for ICU and hospital stay were 1.9±1.3 days (range 1-11 days) and 9.6±2.5 days (range14-21 days), respectively.

Immediately after the operation, SR was restored in 85.3% of the patients (*P*=0.50). Of those patients, many had recurrence of atrial tachyarrhythmias within the first or second week after the Maze procedure. The New York Heart Association (NYHA) functional class had improved by the last follow-up as compared with the preoperative class in all patients (2.3±1.4, *P*<0.0001)

Following discharge from the hospital, patients' follow-up was performed as outpatient clinic examinations and the average follow-up period of patients was 18±3 months. SR was present in 28 (70%) patients and AF was identified in 12 (30%) patients in their 3^rd^ month control examination. While the incidence of SR was 85.3% on the first postoperative day, it was 80% during discharge and 71% in the 1^st^ year follow-up examination. In terms of functionality, NYHA values showed improvement in the postoperative period. Since AF was identified in the 6^th^ month follow-up in three patients, electrical cardioversion was performed; in two patients, the rhythm returned to normal SR and because AF persisted, one patient was discharged by planning outpatient treatment.

## DISCUSSION

In patients with chronic AF, the rate of returning to SR following mitral valve operation is under 10%^[5]^. There has been a demand for less invasive procedures, such as manipulation only in the pulmonary veins and intraoperative ablation of atrial walls with alternative energy sources (cryoablation, microwave, RF, laser, and ultrasound), and thoracoscopy procedures using RF and ultrasound catheters from the epicardium in on-pump surgeries^[6]^. With ensuring SR and effective atrial contraction, myocardial remodeling and heart failure due to tachycardia are prevented^[1]^. Forlani et al.^[7]^ reported that ensuring SR increased the survival rate and decreased the morbidities significantly in patients. For this reason, in patients having chronic AF rhythm, if cardiac surgery is planned, addition of ablation treatment is suggested. On the other hand, AF, which is present approximately in 5% of patients with coronary artery disease, may continue following recovery of myocardial ischemia^[8]^. Since AF was shown to reduce the long-term survival rate following coronary bypass procedures, surgical ablation is recommended in these patients^[9]^. AF ablation has shown a rapid progress by utilization of various energy sources such as RF, microwave, laser, ultrasound, and cryoablation. Nevertheless, the success rates vary between 76% and 92% in surgical ablation methods excluding Maze^[10]^. There are many studies comparing the RF ablation treatment of AF with patients in whom this procedure was not performed. Khargi et al.^[11]^ reported that at the end of the first year, 80% of patients in whom RF was performed and 20% of patients in whom only mitral valve repair was performed had SR. Melo et al.^[12]^ found that, in the first month, SR was present in 7% of patients in whom RF ablation was not performed. Jessurun et al.^[13]^ found sinus, paroxysmal AF and chronic AF rhythms preoperatively in 162 patients, 71% of patients with SR, 34% of patients with paroxysmal AF, and 4% of patients with chronic AF were in SR. Brick & Braile^[6]^ analyzing studies with immediate results (n=5), the percentage of return to SR ranged from 73% to 96%, while those with long-term results (n=20) (from 12 months on) ranged from 62% to 97.7%. Even with postoperative antiarrhythmic drug treatment and electrical cardioversion, returning to SR was found below 25% in the long-term follow-up^[14]^. Early postoperative AF originates from delayed healing processes of atrial lesions, inflammatory processes related to the procedure, and tiny macro re-entries, which have responded perfectly to the antiarrhythmic treatment; for this reason, amiodarone treatment with a dose of 200 mg/day is used during the first postoperative three months.

One of the most important purposes of returning the patients who were in AF preoperatively to SR is to provide atrial contraction and atrioventricular electromechanical synchrony, and additionally to reduce the risk of cardiac embolus. Waldo et al.^[15]^ showed that thromboembolic complications were met with less frequency in AF patients who underwent Cox-Maze operation, when compared to the patients who did not undergo this operation. In our study, also, no thromboembolic event was observed in any of the patients.

It is suggested that the variations observed in results are related to patient characteristics, ablation lines, and technical variables. In some patients, permanent pacemaker may be required following ablation procedure. The frequency of requirement for pacemaker generally varies between 5-10% in publications^[16]^. Permanent pacemaker requirement is considered more frequent in patients having large LA diameter and sick sinus syndrome in the preoperative period. Postoperative drug treatments were mainly for rhythm control. We observed that in patients that SR was acquired, life quality during their daily living improved with medical treatment. The heart rates of patients having AF rhythm were observed to be under control and episodes of disturbing tachycardia were not present.

## CONCLUSION

In conclusion, the early postoperative results of monopolar and bipolar RF ablation in patients undergoing mitral valve surgery were successful in terms of providing atrial transport function and it was observed that these procedures did not lead to additional complications in the postoperative period. Therefore, we suggest that, in patients with AF who are planned to undergo mitral valve surgery, RF ablation for AF is an effective and reliable method for eliminating postoperative risks related to AF and for increasing benefits of surgery.

**Table:** 

Authors’ roles & responsibilities	
AÇ	Conception and design study; realization of operations and/ or trials; analysis and/or data interpretation; statistical analysis; manuscript writing or critical review of its content; final manuscript approval
UK	Analysis and/or data interpretation; final manuscript approval
MC	Final manuscript approval
NB	Final manuscript approval
HK	Final manuscript approval
